# Which Device Is Most Suitable for Measuring Heart Rate Variability in the Field? A Comparative Evaluation of Two Leading Options

**DOI:** 10.1097/JOM.0000000000003479

**Published:** 2025-06-05

**Authors:** Maryline Krummenacher, Mika Tarvainen, Estelle Montet, Michelle C. Turner, Irina Guseva Canu

**Affiliations:** From the Department of Occupational and Environmental Health, Unisanté, University of Lausanne, Lausanne, Switzerland (M.K., E.M., I.G.C.); Department of Technical Physics, University of Eastern Finland, Kuopio, Finland (M.T.); Department of Clinical Physiology and Nuclear Medicine, Kuopio University Hospital, Kuopio, Finland (M.T.); Kubios Oy, Kuopio, Finland (M.T.); Barcelona Institute for Global Health (ISGlobal), Barcelona, Spain (M.C.T.); Universitat Pompeu Fabra (UPF), Barcelona, Spain (M.C.T.); and CIBER Epidemiología y Salud Pública (CIBERESP), Madrid, Spain (M.C.T.).

**Keywords:** heart rate variability, monitoring, sensor, autonomy, safety

## Abstract

**Objective:**

The aim of the study was to compare the Polar H10 and Bittium Faros™ heart rate variability monitors for use in occupational and environmental health research, considering data collection, storage, transfer, resource needs, and limitations.

**Methods:**

A literature review, device testing, and expert interviews were conducted to assess device performance and usability.

**Results:**

Polar H10 is affordable, records RR intervals for up to 20 hours offline, and requires a smartwatch and app for extended storage. Bittium Faros™ is a certified electrocardiogram monitor that records full waveforms, supports long measurements, and enables automatic data transfer. Both devices can be worn with a chest strap, but placement issues may affect data quality. Patch electrodes improve stability but may cause skin irritation.

**Conclusions:**

This assessment highlights key device differences to guide HRV monitor selection for occupational and environmental epidemiological studies.

## Abstract 2

Polar H10, often considered a “gold standard” for heart rate measurement, is a fitness-oriented device suited for nonclinical use with shorter recordings. Bittium Faros™, though costlier, offers medical and functional reliability, extended recording (24+ hours), and advanced data transfer and storage, making it preferable for clinical and field studies.

## Graphical abstract

**Figure:**
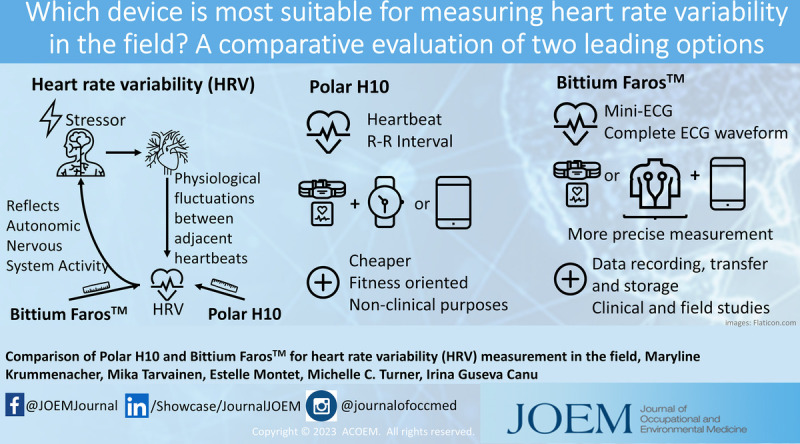

**LEARNING OUTCOMES**

- Be able to critically evaluate the strengths and limitations of the Polar H10 and Bittium Faros™ for HRV monitoring in occupational and environmental health research.
- Be able to select the most suitable HRV device based on data collection, storage, and transfer modalities, as well as practical constraints such as cost, accessibility, and required resources.
- Gain an understanding of the impact of device placement and electrode type on data quality, ensuring optimal measurement conditions for epidemiological studies.

Through the concept of the exposome, public health research integrates all the exposures of an individual in a lifetime, the biological manifestations of those exposures, and how the exposures and effects relate to health.^1–3^ Worksome, which captures the diversity and range of occupational exposures, including chemicals, psychosocial stressors, and physical factors at the workplace, as well as their corresponding biological responses, represents an important contribution to the exposome.^4^ Studying rapidly evolving working conditions enhances the relevance of the exposome framework in occupational research settings and requires new methods and tools to capture these changes and their repercussions for human health.

Heart rate variability (HRV) is increasingly used in experimental and observational studies related to occupational and environmental exposures. The relevance of HRV monitoring has been demonstrated in high-stress occupations among tactical personnel, such as military, police, and firefighters^5^ and in occupations impacted by climate change and energy transition^6^ including in public transit.^7^

HRV reflects the physiological fluctuation in time intervals between adjacent heart beats^8^ and can act as an indicator of body equilibrium to adapt to environmental changes.^9^ HRV reflects the autonomic nervous system activity^10^: the sympathetic activation of the autonomic nervous system leads to a decreased HRV while parasympathetic activation induces an increase of HRV. An optimal level of HRV is associated with health and self-regulatory capacity, as well as adaptability or resilience, reflecting an advantageous balanced autonomic nervous system. Decreased HRV can be indicative of a health problem such as of cardiac,^11^ metabolic^12^ or respiratory disease.^13^ HRV is widely used as a physiological indicator in health research^11^ and can also be used as a predictor of work stress in occupational studies.^14^ Guidelines for HRV parameters, analytical methods and influencing factors have been issued to support high quality recording of HRV.^15^

The most accurate method to record HRV is the use of electrocardiogram (ECG).^9^ This is, however, often impractical and incompatible with the working activities in occupational and research settings. Several wearable HRV measurement devices have been developed and are commercially available, rendering HRV monitoring more accessible. Existing devices have been reviewed with respect to their functional principles as well as validity and reliability of the HRV measurement results compared to ECG.^16–18^ However, none of these reviews systematically assessed safety-related aspects associated with HRV device usage (eg, potential adverse effects) or practical aspects with respect to recording, storing, and use of the HRV monitoring data. The present overview focuses on these aspects with the aim of facilitating the selection of the most appropriate instrument for HRV measurement in the context of occupational and environmental epidemiological field research.

## MATERIAL AND METHODS

To inform the above-mentioned aspects and collect necessary data we used a multilevel approach. First, we scrutinized available literature, and more particularly the systematic and narrative reviews^5,10,16,17,19,20^ in order to identify wearable HRV devices and review the data regarding their measurement validity and precision from the medical point of view (ie, by comparison with the reference method [ECG]). Second, we consulted the website of producers and distributors, as well as technical documentation provided for each of the selected devices. Third, we contacted experts from Swiss and international research centers specialized in HRV measurement. Online and face-to-face meetings were arranged regarding their opinions of the quality and security of data recording, storage, and extraction, existing drawbacks and possible solutions for their management.

## RESULTS

### Selected Devices and their Main Features

According to the available literature, there is an agreement that two devices, the Polar H10 (Polar Electro Oy, Kempele, Finland) and the Bittium Faros™ 180L (Bittium Oyj, Oulu, Finland) are currently the most validated and, consequently, the most recommended as reliable devices for use in field studies.^17,18^

Polar H10 is presented as “a gold standard” for heart rate measurement, mainly due to its validation and popular usage in physical activity settings including in military activities in the field.^21^ It is easily commercially available and user friendly in daily usage. It is used with a chest strap, adapted for usage in water. The sensor needs to be combined with a smartwatch or mobile app supporting HRV data recording. Polar H10 costs around $100 USD, however, additional costs are needed for the smartwatch or the HRV app. Prices of smartwatches vary depending on the model ($350–$850 USD).

Bittium Faros™ is classified as reliable for medical-grade ECG monitoring and adheres to strict regulatory standards (eg, FDA approval or CE marking) for use in clinical environments.^22^ Access to the Bittium Faros™ is typically provided through medical device distributors, healthcare providers, hospitals, and clinics that are authorized to use and prescribe medical-grade equipment. It can also be available via direct sales channels for healthcare institutions or research organizations, subject to regulatory approval in the respective country. Additionally, certain healthcare professionals (eg, cardiologists or medical researchers) can prescribe its use for diagnostic or monitoring purposes.

Bittium Faros™ typically costs between $1000 to $4000 USD for the device itself, depending on the model and features, and the cost may increase depending on the required accessories (chest strap with adaptor and cables for data transfer). The average cost for renting is $100–150 per month and depends on the renting duration with some providers offering daily or weekly rental rates for short-term use. However, the chest strap and the sensor adaptor need to be purchased additionally ($85 for both). It can be worn either using a chest strap or using patch electrodes. The material also comprises a smartphone that allows the automatic transfer of the recorded data to the web-based platform.

### Data Recording and Internal Storage

Polar H10 makes a continuous recording of heart rate and interbeat intervals at 1000 Hz (1-ms resolution). When used together with a Polar smartwatch, an account on the Polar Flow application must be created to store the recordings. Polar H10 allows individuals to start, record, and sync heart rate data with their own devices and apps; however, it lacks research-specific configurations to ensure proper data recording when multiple participants sequentially use the same device in a research experiment. Thus, researchers should activate and pair the Polar H10 with the Polar Beat app, manually starting and stopping each recording to maintain session isolation and prevent data overlap. It is essential to transfer and label each participant’s data immediately after recording, as the H10 can only store one session at a time, creating a risk of data loss if new recordings overwrite previous ones. Consistency in protocol must be strictly maintained by ensuring that participants do not attempt to operate the device independently, as this could lead to pairing conflicts or errors in data segmentation.

Smartwatch measurements are only able to store the beat-to-beat time intervals or RR intervals of the ECG, not the ECG waveform data itself. There are also some apps (eg, HRV Logger, HRV+) that support HRV data recording with Polar H10. In addition, Polar offers a Software Development Kit (SDK) for app developers for enhanced data acquisition from Polar sensors, including the H10. Mobile apps utilizing Polar’s SDK can support online recordings of RR intervals (ie, the time between consecutive R-wave peaks on an ECG, which represent ventricular depolarization), ECG waveform (sampled at 130 Hz), and acceleration data. Additionally, offline recordings of RR data are supported with the H10.

In the offline recordings, the RR data is stored in the internal memory of the H10, which limits the data acquisition to 95,000 RR intervals, equaling ~20 hours of normal daily living. In the online recordings, the selected data channels are streamed in real-time via Bluetooth from the sensor to the app. Online recordings involve the following challenges: first, the range of the Bluetooth is relatively short, the distance between the sensor and the mobile device should not be more than 10 meters; otherwise, the Bluetooth connection is lost leading to loss of data. Another challenge for longer-term recordings is related to background activity restrictions of mobile devices. Unless the recording app has strong enough background processing permissions, the ongoing recording might be stopped by the mobile operating system to save battery and/or optimize functionality of other apps. Therefore, special attention needs to be paid to app permissions and battery optimization settings when running an online measurement with an app supporting Polar SDK. A mandatory use of a paired smartwatch or an app^23^ to store the recorded data collected though the chest strap was identified as a concern by several experts. In studies where HRV should be monitored continuously for several days, secured long-term storage of the data is primordial. However, the need for the paired-smartwatch or associated app can become critical in settings like public transport research where the workers would not be allowed to wear an additional externally visible devices even a smartwatch^24^ or where it could be impossible to install the needed app on the professional tablet, smartphone or computer. However, the main limiting point for the recording duration is the sensor batteries, which must be regularly charged (ie, recordings of up to 6 days are possible without charging the sensor batteries).

Bittium Faros™ uses multiple electrodes, depending on the configuration (eg, 1-lead, 3-lead, or more) to detect electrical potentials, allowing it to capture detailed information about the heart’s electrical activity. The Bittium Faros™ thus records the complete ECG waveform (P wave, QRS complex, and T wave) and provides insights into the heart’s rhythm, conduction pathways, and possible abnormalities (eg, arrhythmias, ischemia). The Bittium Faros™ typically records at sampling rates of 125 Hz to 1000 Hz (1- to 4-ms resolution). Its adjustable sampling rates allow to balance data accuracy with storage and power efficiency, making it versatile for both clinical and research purposes. Indeed, the Bittium Faros™ is capable of continuous, long-term ECG recording for applications like Holter monitoring, which requires stable and accurate ECG data over hours or days. Each rented sensor is directly preregistered in the web portal CardioFlex® (Bittium Medical Suite) and the device provider gives a personal access to this portal to the researcher. This allows individual preregistration of all participants and linking their specific sessions to individual profiles to ensure that each participant’s data is clearly separated and correctly labeled for analysis. CardioFlex® also enables researcher to specify recording parameters (eg, sampling rate, duration, event markers) for each participant beforehand and thus to ensure the consistency of the research protocol by maintaining uniform recording conditions for all participants. Recording sessions can be started and stopped manually or preconfigured via the CardioFlex® system. This creates a potential constraint since trained researchers and a computer are needed to start and stop the recording when using this device but reduces errors during the experiment. The sensor has a long-lasting battery, allowing up to 14 days of recording based on the manufacturer recommendation, as long as the sensor and the smartphone are charged. It is recommended to charge the smartphone every 3–4 days, but, based on observations, it may be safer to charge the smartphone every day, to be sure that it will not be switched off, the latter generating a risk of data loss. The sensor and the smartphone can be easily charged using the same charger. The smartphone allows an automatic data transfer to the Bittium Faros™ analytical platform Cardiac Navigator™ and does not need to be kept close to the device during the day, and it can be left at home for example. This is an advantage for occupational studies since workers do not need to keep an additional smartphone with them. It suffices to put the sensor next to the smartphone at the end of the day for automatic transfer of recorded data.

### External Data Storage

Data recording through Polar H10 and the associated smartwatch are stored on the Polar Flow account that allows global participant data management of all recording. In the context of studies with several participants with ongoing recordings, it is important to note that all data will be transferred in the same Polar Flow account, without participants labels. It is therefore necessary to carefully identify each participant. Ideally, one account per participant should be created. However, each Polar Flow account requires an e-mail address, and one e-mail address can be linked with a maximum of five accounts, creating potential constraints for the research team.

Recorded data from Bittium Faros™ are continuously stored on the Cardio Navigator™ platform, linked with each participant account on CardioFlex® and managed by the researcher. This allows a real-time monitoring of the recording and follow-up of participants and preventing risk of data overlap or loss between participants. After session, the data can be easily assigned to the correct participant and exported for detailed analysis.

### Data Extraction

Stored raw data from Polar H10 can be extracted from the Polar Flow in .csv files. The file contains heart rate and RR interval within a 1-millisecond resolution.

Recorded raw data from Bittium Faros™ can be visualized on the Cardiac Navigator™ dashboard. Data can be also exported in .csv files to perform further analysis. It is necessary to retrieve data from Cardiac Navigator™ and store them externally before starting a new recording with a given device because the data will be overwritten by the latest recording.

### Data Management and Analysis

HRV analysis requires either beat-to-beat RR interval data or continuous ECG waveform data from which RR intervals can be extracted. When raw ECG data is available, it allows researchers to verify the origin of any abnormal RR intervals observed during analysis. For example, clinical verification of abnormal heart rhythms necessitates ECG confirmation. The most important time-domain indices of HRV are based on normal-to-normal beat intervals, originating from sinus node action potentials. Therefore, preprocessing of RR interval data must, at a minimum, involve the identification and correction of abnormal heartbeats using a validated method.^25^ Additionally, preprocessing may include identifying noisy data periods to enhance the overall reliability of the analysis and remove very low-frequency components of HRV to improve the sensitivity of the analysis to short-term changes in RR intervals driven by the sympathetic and parasympathetic nervous systems.^26^

HRV analysis should be conducted using validated methods or software to ensure accuracy and reliability. The results are typically presented as a standardized set of time-domain, frequency-domain, and nonlinear parameters. Kubios HRV Scientific software (Kubios Oy, Kuopio, Finland) works with both Polar H10 and Bittium Faros™, and a free Lite version with limited functionality is also available. Other commercial HRV analysis programs include AcqKnowledge 4.4 (BIOPAC System Inc.) and Nevrokard® (Nevrokard), but also Open Source software such as Artiifact (https://github.com/tobias-kaufmann/ARTiiFACT). Heart Rate Variability Analysis Software (HRVAS) (https://github.com/jramshur/HRVAS) in MATLAB, hrv-analysis in Python (https://github.com/Aura-healthcare/hrv-analysis/blob/master/hrvanalysis/extract_features.py), and RHRV R package (https://cran.r-project.org/web/packages/RHRV/index.html) are available for data processing and/or analysis.

### Undesirable Effects

Only few studies have reported minor undesirable side effects such as mild skin eruption due to the wear of the patch electrodes.^16^ By having tested the ECG device Bittium Faros™, we confirmed that the use of patch electrodes, especially in the context of known plaster-associated allergy can create skin irritation. Some collaborators using the Bittium Faros™ in their ongoing study^27^ shared some preliminary observations in which a few participants also reported mild skin irritation due to prolonged wear (more than 10 days) of the patch electrodes.

Skin irritations were also reported after wearing the chest strap.^27^ For both devices the chest strap should be worn under the chest in order to guarantee an optimal recording of ECG signals. Indeed, dry electrodes in chest straps offer lower conductivity and stability compared to wet electrodes used with the Bittium Faros patches. Moreover, wearing the sensor on the chest strap can be problematic for individuals with concave shape of the sternum, which can interfere with the proper placement or functioning of sensors. Indeed, if the chest strap is moving and not optimally placed, it can create less reliable data. For women, the usage of a HRV monitor on the chest strap can be problematic when wearing a bra, because the lower part of the bra overlaps with the chest strap. This issue is circumvented by using the Bittium Faros™ device with the patch electrodes.

Table 1 summarizes the evaluated features of Polar H10 and Bittium Faros™ to facilitate the decision making.

**TABLE 1:** Comparative Assessment of Logistical and Functional Characteristics of Polar H10 and Bittium Faros™

Characteristics	Polar H10**https://www.polar.com/en/sensors**	Bittium Faros™**https://www.bittium.com/medical/bittium-faros/**
Recorded parameters	Heart rate and RR intervals. Raw ECG data capture is possible but needs a live connection to an external device through Polar’s Bluetooth Low Energy (BLE) Software Development Kit (SDK)	Medical grade ECG: records the complete ECG waveform
Sampling rate	1,000 Hz	125–1,000 Hz (adjustable)
Accessories needed	A chest strap A smartwatch and mobile app for data recording and transfer	A chest strap with adaptor or electrodes A USB cable or a smartphone for data transfer
Maximal memory capacity in offline recording)	95,000 RR intervals (~20 hours)	4GB (~93 days of continuous single-channel ECG recording and ~32 days for 3 channels of ECG data, sampled at 250 Hz with 16-bit resolution)
Maximal battery autonomy	6 days, 3–4 days for the smartphone	14 days for the sensor, 3–4 days for the smartphone
Accessibility for purchase/rent	Online	Through medical institutions or Bittium’s direct sales channel
Purchase cost	~$100 USD	$1,000–$4,000 USD for the device and the smartphone
Additional costs	$350–$850 USD for the smartwatch.	$85 USD for the chest strap with adaptor and cable for data transfer
Rental cost	Cannot be rented	$100–$150 USD/ month Chest strap with adaptor and cable for data transfer need to be purchased separately
Software/app needed for data recording	Polar Beat mobile app on a paired smartphone is necessary for recording HR data. Polar Flow used with a smartwatch or a third-party app is necessary to store RR data.	The sensor comes with a manager software, enabling to setup recording preferences. After the recording data from the sensor can be download in EDF format. CardioFlex® app is helpful to preregister sensors (see below)
Specificities of data recording	A trained researcher should control the app activation and device pairing and manually starting and stopping recording session for each participant. Researchers should ensure that participants do not attempt to operate the device independently.	Sensors preregistered on CardioFlex® Recording parameters can be defined on CardioFlex® A trained researcher and a computer are needed to start and stop the device recording
Software/app/devices necessary for data transfer	A smartwatch with Polar Flow or a smartphone with Polar Beat app and permanent Bluetooth connection	Data is transferred by connecting the sensor directly to a computer via USB
Specificities of data transfer	The sensor and mobile device should be kept close (max 10 m) to not lose Bluetooth connection. The app needs strong enough background permissions, to not be shut down by mobile operating services.	The smartphone allows automatic data transfer to Cardiac Navigator™. It is enough to put the sensor next to the smartphone once a day to transfer the data.
Advantages/disadvantages of data transfer modalities for occupational studies	During the measurement, study participants need to carry either a smartwatch, which they may not be allowed to wear, or a mobile device with an app, which might be impossible to install on professional IT equipment.	Internal memory capacity of the sensor is 4GB Study participants do not need to carry the smartphone during their work shift.
Data storage platform	Polar Flow	Bittium Medical Suite (online platform)
Specificities of data storage	A Polar Flow account can store up to 5 participants data and needs an email address. An e-mail can be linked to max 5 accounts. The participants need to be carefully labeled or have their own account.	Link between each participant’s account on CardioFlex® (Bittium Medical Suite) and Cardiac Navigator™ (analytics environment)
Data available for extraction	RR intervals	Complete ECG waveform
Software/application necessary for data extraction	Polar Flow	Cardiac Navigator™
Extracted file format	Training Center XML (TCX), CSV	European Data Format (EDF), ie, binary file format for storing multichannel time-series data and
Software/applications necessary for data management and analysis	Several commercial and open-sources programs and web-based applications (eg, Kubios HRV; Nevrokard aHRV, RHRV)	Several commercial and open-sources programs and web-based applications (eg, Kubios HRV; Nevrokard aHRV and LT-HRV, Bittium Cardiac Navigator™, and Cardiaoscope™, RHRV)
Undesirable health effects	Potential mild skin irritation due to the chest strap	Potential mild skin irritation due to the chest strap or the electrodes
What could affect data quality	The chest strap moving Participant clothing	The chest strap moving and participant clothing, however, the use of electrode patches avoids this issue.

ECG, electrocardiogram; CSV, comma-separated values; EDF, European Data Format; HR, heart rate; HRV, heart rate variability; IT, Information Technology; LT-HRV, long-term heart rate variability; RHRV, R heart rate variability; RR interval, beat-to-beat time interval in ECG; USB, Universal Serial Bus.

## DISCUSSION

### Main Findings

The Bittium Faros™ meets the technical and regulatory requirements for recording and analyzing the full electrical activity of the heart like an ECG device, while the Polar H10 is a fitness-oriented device that measures RR intervals. Despite its practicality for individual monitoring, convenient for nonclinical purposes, the lack of built-in participant management features in the Polar H10 makes it challenging for structured research involving multiple subjects without meticulous supervision. For clinical and field studies, Bittium Faros™ offers medical and functional reliability, particularly with respect to data recording, transfer, and storage. Moreover, the patch system (with 3 or 5 electrodes) allows for a more precise capture of the electrical signals from the heart, as it improves skin contact and reduces noise and motion artifacts, which are common with chest straps during exercise or movement.

### Study Strength and Limitations

We believe that this study is timely and addresses practical and concrete aspects of current research in occupational and environmental health. The chosen methodology intentionally diverges from a systematic review, scoping review, or mapping study,^28–30^ as the available literature and existing reviews do not cover the logistical aspects of handling portable HRV measurement devices in field studies, nor the challenges related to data recording, storage, retrieval, and processing. Our methodology builds upon existing research, particularly the most recent systematic reviews,^16–18^ to identify the most validated and widely used devices. It then integrates multisource data and expert opinions—including those of researchers who use these devices and engineers who develop data processing software—to compare the preselected devices based on a systematic evaluation of key properties deemed essential for device selection in research. Thus, by design, this study closely resembles a mixed study approach,^31^ where triangulation allows for the integration of qualitative data, collected through interviews, and quantitative data. This approach has already been used in occupational health research and has proven effective in addressing practical research questions.^32–34^

The expert interviews were not recorded or analyzed using qualitative methodologies^35^; rather, they were conducted pragmatically to verify and confirm information obtained from manufacturer and distributor websites for both the devices and their analytical software. The interviewed experts constituted a convenience sample, as probability sampling was not feasible within the study’s timeline. Despite these limitations, we believe that the systematic comparison approach, based on triangulation of collected data, produces accurate and reliable results, which are valuable for research in occupational and environmental medicine.
